# The New Health Commissioner

**Published:** 1910-04

**Authors:** 


### The New Health Commissioner

DR. FRANCIS E. FRONCZAK was appointed Health Commissioner of Buffalo by Mayor Fuhrmann on March 27, 1910, to fill the vacancy occasioned by the death of Dr. Ernest Wende, which occurred February 11, 1910. The mayor has not taken this action with unseemly haste. He has paid due regard to the memory of Dr. Wende in delaying the appointment for six weeks. Moreover, in our view he has further observed the proprieties of the occasion by promoting the deputy to the place so long and ably filled by his chief. In this the Mayor has followed the spirit of the Civil Service, which always advocates promotion where ability justifies such action.

In these remarks we would not reflect in the least upon the candidates for the place whose names have appeared in the newspapers in that relation, many of whom we regard as friends and all as physicians of excellent professional standing. None of them however, has served in the office, as has Dr. Fronczak who performed the duties thereof for two years more or less, that is to say while Dr. Wende was incapacitated from ill health for active duty. Other things being equal, this fact should strengthen Dr. Fronczak's claim to the place, and we have no doubt the Mayor gave it great consideration in making the appointment.

The announcement was at once made by Dr. Fronczak that he had chosen Dr. Arthur C. Schaefer, for deputy commissioner. This selection, too, is in accordance with the spirit of the civil service plans, Dr. Schaefer having been for sometime before his promotion a school medical inspector in the department of health. The terms of both appointees will expire December 31, 1911, the date of expiration of Dr. Wende's term. The full term of health commissioner is five years and the annual salary is $4,000.

Francis Eustace Fronczak was born in Buffalo, September 20, 1874. His preliminary education was obtained in St. Stanislaus's school and in 1887 he entered the academic department of Canisius College. In 1894 he had earned the degree of bachelor of arts and the following year the A. M. degree from Canisius was conferred upon him. He then entered the medical department of the University of Buffalo graduating therefrom in 1897. The following two years found him in the law school, but his increasing practice made such demand on his time that he did not take the final examinations in the law. In college and university, Dr. Fronczak supported himself largely by work done for local newspapers. In 1898, Mayor Diehl appointed him to the Civil Service Commission and he served until 1903, when Mayor Knight removed the board. In November, 1905, he was appointed deputy health commissioner to Dr. Wende and took office on January 1, 1907, serving continuously since that time at a salary of $2,000.

---

Dr. C. Wardwell Stiles, the discoverer of the American hookworm in the South, whose reports on the subject induced John D. Rockefeller to donate $1,000,000 for the eradication of the disease, has just made a report to Surgeon General Walter S. Wyman, of the public health and marine hospital service, on an inspection of cigar factories in and around Tampa.

Dr. Stiles says that unless the sanitary conditions on the outskirts of Tampa are improved, Florida may have two forms of hookworm to exclude,—the American and the Old World form. The latter form, he fears, may be introduced under favorable condition by Spanish and Italian workers. Dr. Stiles suggests that the readers employed in the tobacco factories to relieve the monotony of the work, might be utilised to acquaint the workers with the disease. The material to be written in popular style, to be read to the workers.

"Most of the deaths in the United States are caused either by suicide or homicide," said Dr. Harvey Wiley, the Government food expert, in a lecture before Cornell students at Ithaca, March 24, 1910.

"The majority of the murders are committed by society and by the environment in which people live. A good many people die from preventable diseases.

"It is a crime to have a cold. If you breathe fresh air all the time you wouldn't get any. They don't have them at the North Pole. I get mine by riding in Pullmans, where less attention is paid to ventilation than to plush and mural paintings."

---

In the *Boston Medical and Surgical Journal,* announcement is made of the discovery by Dr. John J. Hurley of Boston of a new method of producing conscious anesthesia, both bloodless and painless, in human beings, which promises a world-wide revolution in surgery, especially that of the brain and head. It differs materially from the recently tried conscious anesthesia of Dr. Joseph.

Hurley's method consists of an injection of a solution of cocaine, adrenalin and salt solution, beneath the periosteum.

This method has been demonstrated and accepted at the Massachusetts eye and ear infirmary.

The patient was a woman 40 years old, who was admitted to the hospital for the removal of the ossicles of the ear. Dr. E. A. Crockett, of Boston, performed the operation, after Dr. Hurley had anesthetised. The operation was absolutely painless and bloodless, according to the report.

---

Hornell claims the distinction, justly too, we have no doubt, of having established the first municipal hospital in the state of New York for the care of tuberculosis patients of New York. The building was opened for the reception of patients March 25, 1910. It is the intent of the board of health to have all cases of tuberculosis sent to this hospital that they may be properly isolated and the danger of contagion diminished or obviated.

---

Why the quack prospers is well illustrated by a writer in the Washington *Herald.* The regular doctor said to the other, "How is it that you, without education, skill, or the least knowledge of medicine, are able to live in the style you do? You have your town house, your carriage, your motor car and your country house, while I can little more than pick up a bare subsistence."

The quack, so the story goes, laughed good-naturedly.

"Look here," said he. "How many people do you think have passed us on this street here since you asked that question?"

"Well," said the other, "about one hundred."

"And out of that hundred how many do you think possess good common sense?"

"Possibly one," was the reply.

"Well," said the quack, "that one comes to you, and I take care of the ninety-nine."

---

The total receipts of the sale of Red Cross stamps for the relief of tuberculosis in this city amounted to $5,480.55 of which 20 per cent. was remitted to the National Red Cross Association and the balance, $4,472.15, is to be used for the day camp in Buffalo.

---

Dr. Ernst J. Lederle, the new head of the New York Health Department, is not a physician, but is a Doctor of Philosophy of Columbia University. He was born on Staten Island forty-four years ago. He was educated at New York University and Columbia, where he specialised in chemistry. He served as assistant chemist for seven years and a chief chemist for five years in the Health Department prior to his appointment as Health Commissioner by Mayor Low, in 1902. Commissioner Lederle succeeded Dr. Darlington as Commissioner of Health, by appointment of Mayor Gaynor, soon after he took office.

